# Acute Systemic Inflammation is Unlikely to Affect Adiponectin and Leptin Synthesis in Humans

**DOI:** 10.3389/fcvm.2015.00007

**Published:** 2015-03-05

**Authors:** Mattias Ekström, Stefan Söderberg, Per Tornvall

**Affiliations:** ^1^Cardiology Unit, Department of Medicine, Karolinska University Hospital, Solna, Sweden; ^2^Cardiology, Department of Public Health and Clinical Medicine, Umeå University Hospital, Umeå, Sweden; ^3^Department of Clinical Science and Education, Karolinska Institutet, Södersjukhuset, Stockholm, Sweden

**Keywords:** adiponectin, leptin, inflammation, plasma, RNA

## Abstract

Adipose tissue (AT), classically thought to be merely an energy store, has been shown to produce inflammatory and metabolically active cytokines. Recently, adiponectin and leptin, adipokines primarily synthesized by adipocytes, have attracted considerable attention because inflammation has been suggested to modulate adipokine levels. However, the regulation of adiponectin and leptin is complex and the knowledge about their synthesis within the early onset of inflammation is poorly understood. The aim of this study was to investigate if the synthesis of adiponectin and leptin is affected during the early phase of an acute systemic inflammation. Eighteen healthy subjects were allocated to vaccination against *Salmonella typhi* or to a control group, and adiponectin and leptin concentrations measured in plasma during 24 h. Nine patients, without markers of inflammation, undergoing open heart surgery were investigated before and after the operation by analysis of plasma levels and AT gene expression of adiponectin and leptin. Plasma interleukin (IL)-6 concentrations were measured in both cohorts. Plasma levels of IL-6 were doubled after vaccination and increased 30-fold after open heart surgery. Plasma levels of adiponectin and leptin were unchanged after vaccination whereas adiponectin and leptin tended to decrease after surgery. The gene expression of adiponectin and leptin was unaltered in omental and subcutaneous AT after surgery. Despite the use of two models of stimulated *in vivo* systemic inflammation, we found no evidence of an early regulation of adiponectin and leptin synthesis, indicating that these two adipokines are not key elements in an acute systemic inflammation in humans.

## Introduction

Adipose tissue (AT), classically thought to be merely a storage place for energy, has been shown to produce inflammatory and metabolically active cytokines. Chronic activation of innate immune responses within AT characterizes development of insulin resistance and atherosclerosis (1). Recently, adiponectin and leptin, two adipokines that primarily are synthesized by adipocytes, have attracted considerable attention because inflammation has been suggested to modulate adipokine levels. However, the regulation of adiponectin and leptin is complex and the knowledge about their synthesis within the early onset of inflammation is poorly understood.

Acute inflammatory effects on AT are, in this context, of particular interest because of the role of adipokines in the development of atherosclerosis.

Adiponectin decreases with increasing obesity and has been described as anti-atherogenic including its positive effects on insulin sensitivity, whereas leptin is involved in the central regulation of appetite (1–3). A rapid weight loss in obese individuals decreases plasma levels of leptin while adiponectin do not change (4). However, a longer period of weight loss and lifestyle changes increases adiponectin plasma concentrations (5). Chronic kidney disease is not only associated with an on-going chronic inflammation in AT (6) but also associated with hyperadiponectinemia. Unexpectedly, high, rather than low, concentrations of adiponectin predict mortality. However, the role of adiponectin in a uremic milieu is complex and the unfavorable effect might not be explained only by a direct effect of adiponectin, but rather a consequence of concurrent processes as wasting/malnutrition or volume and salt overload, which may increase adiponectin levels (7).

Despite that plasma levels of leptin were increased and positively correlated with proinflammatory cytokines during sepsis (8) and further associated with increased survival of sepsis (9), few studies have addressed the influence of innate immunity on adiponectin and leptin in humans.

Previously, two studies showed that leptin but not adiponectin levels in plasma increase after open heart surgery (10) and lipopolysaccharide (LPS) injection (11), respectively. After LPS treatment, only adiponectin RNA expression decreased in subcutaneous AT. Another study showed no effect of inflammation, caused by subarachnoidal hemorrhage, on adiponectin and leptin RNA in subcutaneous AT (12).

The present study is a sub-study of two earlier studies where acute inflammation was induced by vaccination (13) and open heart surgery (14), respectively, with the objective to investigate if innate immunity stimulates the synthesis of adiponectin and leptin. Vaccination against *Salmonella typhi*, previously shown to cause endothelial dysfunction and to activate coagulation (15–17), is a safe model without any major side effects, which activates the nuclear factor (NF)-κB regulatory pathway and results in two- to fourfold increased plasma levels of IL-6 (13, 15). Open heart surgery with cardiopulmonary by-pass (CPB) is one of the strongest models of induced inflammation found in daily clinic. It results in an extensive acute systemic inflammation with 30- to 40-fold increase in plasma levels of IL-6 after surgery (14, 18). The activation of an acute phase response due to open heart surgery and CPB is a complex process and possibly there are different triggers, as the surgical trauma itself and/or blood contact with non-physiological surfaces of the CPB system (19). However, we have shown that the acute phase response, induced by open heart surgery with CPB, stimulates AT to produce an innate immune response due to up-regulation of the NF-κB regulatory pathway (14).

The use of these two models of inflammation, both acting on AT through activation of the NF-κB regulatory pathway (13, 14), with a focus not only on plasma levels but also on mRNA in both omental and subcutaneous AT will extend our knowledge of adiponectin and leptin regulation. The objective of the present study was to determine if adiponection and leptin are regulated by the NF-κB regulatory pathway.

## Materials and Methods

### Subjects

Eighteen healthy volunteers (16 men and 2 post-menopausal women) with a median age of 60 (49–67) years and BMI of 26.8 (19.8–38.8) were included in the vaccination study. Their basic characteristics have been described in detail (13) and are in brief presented in Table 1. Every second study subject was vaccinated against *Salmonella typhi* (Typhim Vi, Sanofi Pasteur MSD, Sweden), whereas the remaining subjects served as controls. Subjects arrived at 7 a.m. to the Karolinska University Hospital after fasting overnight. After vaccination and/or first blood sample, participants had a light breakfast including a sandwich with cheese and a cup of coffee or tea with sugar and milk as preferred.

**Table 1:** **Basic characteristics**.

Variable	Vaccination study		Open heart surgery study *n* = 9
	Vaccinated *n* = 9	Controls *n* = 9	
Age, years	59 (56–66)	60 (49–67)	65 (43–85)
Sex (men/women)	8/9 (89%)	8/9 (89%)	9/0
Current smokers, *N*	2 (22%)	1 (11%)	1/9 (11%)
Former smokers, *N*	5 (56%)	4 (44%)	4/9 (44%)
Body weight, kg	93 (60–127)	90 (64–109)	79.9 (62.5–92.7)
BMI, kg/m^2^	25 (21–38.8)	28.2 (19.8–32.5)	27.7 (21.1–32.4)
CPB, min	NA	NA	98 (50–221)
Time between sample 1 and 2, min	NA	NA	125 (90–285)
History of Diabetes	NA	NA	1/9 (11%)
**Current medication**
Acetyl salicylic acid	NA	NA	7/9 (78%)
Beta blocker	NA	NA	2/9 (22%)
ACEi	NA	NA	2/9 (22%)
ARBs	NA	NA	2/9 (22%)
Calcium antagonists	NA	NA	3/9 (33%)
Diuretics	NA	NA	2/9 (22%)
Nitrates	NA	NA	4/9 (44%)
Statins	NA	NA	8/9 (89%)

*Data presented as median (min and max values), numbers and percent*.

*No differences were found between vaccinated and controls in the vaccination study*.

*ACEi, angiotensin converting enzyme inhibitor; ARBs, angiotensin receptor blockers; BMI, body mass index, CPB, cardiopulmonary bypass, NA, data not applicable*.

Nine patients undergoing open heart surgery study were studied. They were eligible if they were planned for elective coronary artery by-pass surgery and/or aortic or mitral valve replacement according to a standard surgical procedure at the Department of Thoracic Surgery at the Karolinska University Hospital, Solna, Sweden. Patients were excluded if they had unstable coronary artery disease or were treated with corticosteroids. All patients were male with a median age of 65 (43–85) years and a BMI of 27.7 (21.1–32.4). All patients underwent AT biopsies and blood sampling before and after cardiopulmonary bypass (CPB) as described (14). Basic characteristics are presented in Table 1.

All subjects provided written informed consent to participate in the study and the study protocol was approved by the Ethics Committee of Karolinska Institutet.

### Plasma analyses

Blood sampling before and after vaccination and open heart surgery, respectively, has been described in detail (13, 14). In brief, in the vaccination study venous blood samples were obtained after 0, 4, 8, 12, and 24 h, without an indwelling venous catheter. In the open heart surgery study, the first blood sampling was made after approximately 30–40 min of surgery, before start of CPB. The second blood sampling was made after the CPB had been turned off with a median time of 125 (90–285) min between the paired samples. The blood samples in the open heart surgery study were obtained from an indwelling radial artery catheter. All blood samples were collected in vacutainer ethylendiamid tetraacetic acid (EDTA) tubes and centrifuged in room temperature; where after plasma was separated and stored at –80°C.

Plasma levels of adiponectin and leptin were analyzed in duplicates using a double-antibody radioimmunoassay (RIA) (Linco, St. Louis, MO, USA). Coefficient of variation (CV) for adiponectin was 15.2% at low (2–4 μg/mL) and 8.8% at high (26–54 μg/mL) levels. CV for leptin was 4.7% at both low (2–4 ng/mL) and high (10–15 ng/mL) levels.

Plasma levels of interleukin (IL-6) in the vaccination study were determined in duplicates using a high sensitive ELISA (R&D Systems, Minneapolis, MN, USA) with an intra-assay CV of 9.5%. Plasma levels of IL-6 in the open heart surgery study were analyzed in duplicates using one Quantikine Human IL-6 Immunoassay plate (R&D Systems) with an intra-assay CV of 10.2%.

### Adipose tissue biopsies

Paired AT biopsies of approximately 1 cm^3^ were taken from nine patients, whereof both omental and subcutaneous AT biopsies from six patients, only omental AT biopsies from one patient and only subcutaneous AT biopsies from two patients. The AT biopsies were collected at the same time as the paired blood samples, before institution of CPB and at 15–20 min after removal of the aortic cross-clamp when the patient had been weaned off CPB. The omental AT biopsies were taken through a small opening to the abdomen in the bottom of the wound and the subcutaneous AT biopsies were taken deeply from the side of the median sternotomy incision.

### Gene expression studies

The protocol for total RNA and cDNA preparation has been described in detail (14). To investigate which housekeeping gene to use, cDNA from omental AT from four subjects was analyzed using a TaqMan Human Endogenous Control Plate (Applied Biosystems, Foster City, CA, USA). To analyze AT gene expression, cDNA was mixed with TaqMan^®^ Universal PCR master Mix (Applied Biosystems) according to the manufacturer’s instructions. RT-PCR was made using a custom made Low Density Array (Applied Biosystems) with Adiponectin (Hs00605917_m1) and Leptin (Hs00174877_m1) as target genes and cyclophilin A (Hs99999904_m1) as an endogenous control gene, with a RT-PCR protocol according to the manufacturer’s instructions. Relative quantification of gene expression was calculated with cyclophilin A as the house keeping gene using the first biopsy in every paired analysis as a reference. Cyclophylin A demonstrated stability during inflammation in the endogenous control plate experiment described above with a similar cycle threshold value (Ct-value) to the gene of interest.

### Statistics

The present study had an 80% power (*p* < 0.05) to detect a difference of 7.4 and 5.6 μg/mL in plasma levels of adiponectin and leptin, respectively. Data are presented as median (min–max), mean ± SD or numbers (percent). Differences between continuous variables have been analyzed using Mann–Whitney *U*-test, Wilcoxon signed-rank test or mixed linear models. The significance level was specified at *p* < 0.05.

## Results

Plasma IL-6 levels after vaccination and open heart surgery have been reported (13, 14). Eight hours after vaccination, median plasma levels of IL-6 were 2.0 (0.7–29.6) pg/mL compared to 1.2 (0.4–7.0) pg/ml in the control group (*p* = 0.02), while open heart surgery resulted in an increase in plasma levels of IL-6 from 3.0 pg/mL (1.9–5.9) to 79.8 pg/mL (31.5–190.8) (*p* = 0.008).

**Figure 1:**
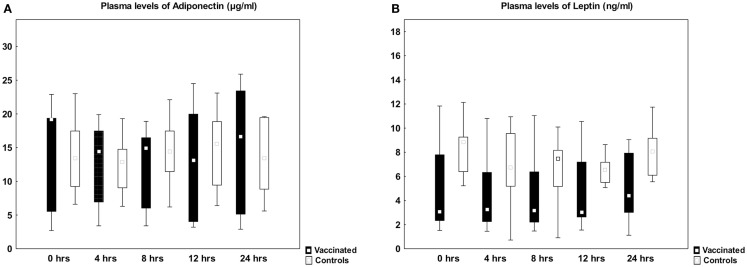
**Plasma levels of adiponectin and leptin before and after vaccination**. Box plots of plasma levels of adiponectin **(A)** and leptin **(B)** in healthy subjects vaccinated against *Salmonella typhi* (*n* = 9, black boxes) and controls (*n* = 9, open boxes). There was no difference between the two groups using mixed linear models. Median, box: 25–75%, whiskers: non-outlier range.

Plasma levels of adiponectin and leptin were unaltered after vaccination, showing similar levels as in the control group (Figure 1). Plasma levels of adiponectin did not change (7.9 ± 4.6 vs. 6.2 ± 2.7 μg/mL, *p* = 0.11) whereas leptin decreased from 8.8 ± 4.4 to 7.3 ± 4.1 μg/mL (*p* = 0.04) after open heart surgery.

**Figure 2:**
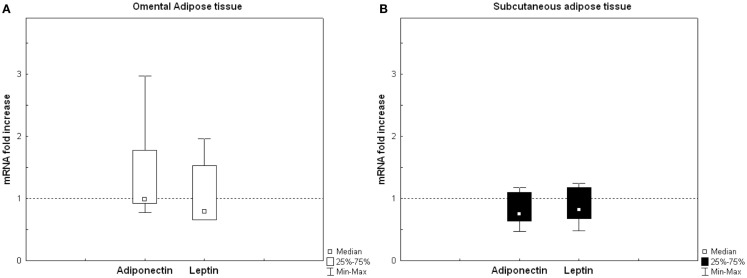
**Relative adipose tissue RNA gene expression after open heart surgery expressed as change from baseline**. Relative quantification (RQ) of adiponectin and leptin in **(A)** omental adipose tissue (AT) and **(B)** subcutaneous AT. RQ levels expressed in relation to the house keeping gene cyclophilin A, using the first biopsy in every paired analysis as a reference (dotted line = no change). Omental AT (*n* = 7) and subcutaneous AT (*n* = 8). There were no differences in gene expression of adiponectin or leptin in neither omental, nor subcutaneous AT using Wilcoxon signed-rank test.

The relative RNA gene expression after open heart surgery, expressed as change from baseline was analyzed in both omental and subcutaneous AT biopsies. Neither adiponectin nor leptin RNA from omental AT did change after open heart surgery. Similar results were obtained for subcutaneous AT (Figure 2).

## Discussion

The results of the present study showed that plasma levels of adiponectin and leptin were unchanged after vaccination whereas adiponectin and leptin tended to decrease after surgery. The gene expression of adiponectin or leptin was unaltered in both omental and subcutaneous AT after surgery. Taking into account that two validated methods of stimulated systemic inflammation were used (15, 18) and that both plasma and gene expression of two AT deposits were examined, it is highly unlikely that adiponectin or leptin synthesis is affected during the early phase of an acute systemic inflammatory response.

When circulating levels of adiponectin were investigated in patients with acute myocardial infarction, a fairly mild stimulus to inflammation, adiponectin was found to decrease after 24 h (20). In our study, we did not find any differences in plasma levels of adiponectin or leptin up to 24 h after vaccination. One possible explanation is that vaccination, that resulted in increased gene expression of tumor necrosis factor (TNF) in peripheral blood mononuclear cells after 4 h and doubled plasma levels of IL-6 after 8 h (13), might have been too weak as a model of inflammation. We have previously reported on a very rapid onset of a strong innate immune response in AT following systemic inflammation induced by open heart surgery, i.e., IL-6, IL-8/CXCL8, and E-selectin showed a 100-fold increase in mRNA expression in omental AT and in subcutaneous AT, these genes increased even more, up to 200- to 400-fold (14). Therefore, we investigated adiponectin and leptin in plasma and on a gene expression level in AT before and after surgery. In plasma, adiponectin and leptin tended to decrease, an effect that certainly is dependent on plasma volume overload (21). In support for unchanged plasma levels, we could not find any change in RNA of these adipokines neither in omental nor in subcutaneous AT after surgery. The results are in accordance with a previous study on open heart surgery investigating epicardial and subcutaneous AT (10). The present study extends these findings to omental AT. In contrast, one recent study (22) showed that gene expression of adiponectin decreased after surgery. However, in that study all patients had preexistent inflammation due either to inflammatory bowel disease or malignancy making interpretation of the effects of acute systemic inflammation difficult.

There are also some recent human studies that have focused on both adiponectin and leptin synthesis in relation to acute inflammation with other stimuli. Anderson and coworkers (11) showed that leptin levels increased in plasma after LPS injection but adiponectin levels remained unchanged. On a gene expression level, only adiponectin was affected with suppressed expression in subcutaneous AT, whereas there was only a trend toward increased gene expression of leptin. The study by Jernås and coworkers (12) showed no effect of inflammation, caused by subarachnoidal hemorrhage, on adiponectin and leptin RNA gene expression in subcutaneous AT. The reason for the discrepant results is not clear but methodological issues have to be considered. The stimulus to inflammation was different between the studies. Anderson and coworkers used LPS whereas we (in the open heart surgery study) and Jernås and coworkers used tissue damage to stimulate innate immunity. All of these are strong stimuli but might activate different subsets of cells, including macrophages in AT. It can be argued that the inflammatory stimulus on AT was similar in our study compared with the study by Anderson and coworkers since we had a similar strong gene expression of IL-6. Also the time-frames were different with the possibility that we, in the open heart surgery study, investigated gene expression in AT too early (approximately 2 h) whereas Jernås and coworkers investigated gene expression too late (days). Finally, there is a possibility that the gene expression results in the study by Anderson and coworkers might be false due to up-regulation of β-actin by inflammation rather than down-regulation of adiponectin. In our study, we also tested the results using other house-keeping genes than cyclophilin All without changing the negative result (data not shown).

To the best of our knowledge there are no *in vitro* studies on the effects of LPS on human adipocytes regarding adiponectin. When leptin synthesis has been studied in cultured human subcutaneous adipocytes it has been demonstrated that TNF attenuated RNA gene expression but in contrast, induced an increased release (23). Furthermore, another *in vitro* experiment by Bruun and coworkers have shown that the proinflammatory cytokines IL-1β and TNF both decreased leptin gene expression and protein production but interestingly IL-1β was found to elicit an early release of leptin (24). The results of these two *in vitro* studies suggest a pre-formed pool of leptin in human AT with a paracrine regulation to which IL-1β and TNF could be important key regulators. However, this pool has not yet been identified and it is not clear whether human adipocytes stimulated *in vitro* adequately represent the situation on a tissue level *in vivo*. In the present study, we could not find any support for secretion of a preformed pool of leptin stimulated by acute inflammation.

The main limitation of the present study is the low number of subjects investigated. In the plasma determination part of the study, a mixed linear model was used to compensate for the number of subjects investigated. Furthermore, in the gene expression experiment, we used paired AT biopsies, from two different AT sites (omental and subcutaneous), which minimize the possible inter-individual variation. Another limitation is the time-frame for AT biopsies with approximately 2 h between the samples. An extended time between AT biopsy sampling might have resulted in a positive result. An argument against this is that inflammatory markers such as IL-6 increased more than 100-fold (14).

Both models of stimulated inflammation used in this study activate inflammation through the NF-κB pathway and interestingly, none of the models were shown to have any influence on the synthesis of adiponectin or leptin, which indicates that the NF-κB pathway is not involved in the regulation of these two adipokines in an acute-phase response. This is also supported by a recent study by Diez and coworkers who showed that leptin was not found to act as an inflammatory reactant but more as a marker of nutritional status in patients with pneumonia (25).

In conclusion, despite the use of two models of stimulated *in vivo* systemic inflammation we found no evidence of an early regulation of adiponectin and leptin synthesis, indicating that these two adipokines are not key elements in the early phase of an acute systemic inflammation in humans.

## Author Contributions

The authors have contributed as follows: ME and PT initiated and designed the study. ME and SS performed the experiments. ME and PT drafted the manuscript. All authors contributed to the interpretation of the results and revised the manuscript critically for important intellectual content and approved the final version of the manuscript.

## Conflict of Interest Statement

The authors declare that the research was conducted in the absence of any commercial or financial relationships that could be construed as a potential conflict of interest.

## Abbreviations

ACE, angiotensin converting enzyme inhibitor; ARB, angiotensin receptor blocker; AT, adipose tissue; BMI, body mass index; cDNA, complementary deoxyribonucleic acid; CPB, cardiopulmonary bypass; CV, coefficient of variation; EDTA, ethylendiamid tetraacetic acid; ELISA, enzyme-linked immunosorbent assay; IL, interleukin; LPS, lipopolysacharide; NA, non-applicable; NF-κB, nuclear factor κB; RIA, radioimmunoassay; RQ, relative quantification; RT-PCR, real-time polymerase chain reaction; TNF, tumor necrosis factor.
